# Kounis syndrome type I induced by an intramuscular injection of diclofenac: A literature review based on a case report

**DOI:** 10.1002/ccr3.9198

**Published:** 2024-07-21

**Authors:** Pouya Ebrahimi, Roozbeh Nazari, Nahid Senobari, Seyedeh Maryam Mousavinezhad, Delaram J. Ghadimi, Hamidreza Soleimani

**Affiliations:** ^1^ Cardiology Department, Tehran Heart Center, Cardiovascular Disease Research Institute Tehran University of Medical Sciences Tehran Iran; ^2^ Department of Cardiology, Modarres Hospital Shahid Beheshti University of Medical Sciences Tehran Iran; ^3^ Department of Cardiology, School of Medicine Ahvaz Jundishapur University of Medical Sciences Ahvaz Iran; ^4^ Radiology Department, School of Medicine Shahid Beheshti University of Medical Sciences Tehran Iran

**Keywords:** acute coronary syndrome, allergic reaction, cardiovascular disease, coronary angiography, Kounis syndrome, NSAIDs

## Abstract

**Key Clinical Message:**

The history of any allergy to the medications should be asked by physicians before administration of the medication. The coincidence of allergic and ACS symptoms after a short time of drug administration might be an indicator of Kounis syndrome. Allergic and coronary symptoms should be considered and treated.

**Abstract:**

Ischemic heart disease is still the leading cause of death worldwide. Some medications, including NSAIDS and antibiotics, can cause allergic reactions with cardiac manifestations due to spasms of the coronary arteries. In this case, we present a patient with chest pain syndrome due to a hypersensitivity reaction caused by an intramuscular (IM) diclofenac injection. The patient was a 51‐year‐old male who presented to the emergency department complaining of retrosternal chest pain, breathlessness, and pruritis that started half an hour after an IM diclofenac injection he had because of low back pain. The allergic symptoms subsided with an antihistamine injection, but chest pain and dyspnea remained stable. He was admitted due to the presence of ST‐segment depression in leads II, III, and AVF and underwent percutaneous coronary angiography, which was normal. The patient was discharged with the diagnosis of Kounis syndrome, and he had an uneventful follow‐up 1 year later. Kounis hypersensitivity‐associated acute coronary syndrome, especially type I variant coronary spasm due to endothelial dysfunction is a type of acute myocardial syndrome. The following report describes an uncommon case of anaphylaxis‐associated Kounis type I syndrome manifesting ST‐segment changes in a male patient following an intramuscular injection of diclofenac.

## 1 INTRODUCTION

Ischemic heart disease is defined as an insufficient blood supply to the local part of the myocardium following abnormally narrowed or blocked blood vessels responsible for blood supply to the area.^1^ Based on several studies, heart disease has been the most common cause of death since 2021, especially in patients with predisposing medical conditions such as diabetes.^2^, ^3^, ^4^ The most common presentation of IHD is chest pain (angina), shortness of breath, fatigue, sweating, and palpitations.^5^, ^6^ However, sometimes, symptoms of IHD can be seen due to nonobstructive causes and in patients without significant risk factors or a history of IHD.^7^

Kounis syndrome is a multidisciplinary condition that affects the circulatory system and manifests as spasm or thrombosis.^8^ Kounis and Zavras described this syndrome for the first time in 1991,^9^ can cause acute myocardial infarction (MI) with exposure to irritants such as medications, food, or environmental triggers. These cardiac symptoms might be accompanied by or without allergic and anaphylactoid reactions.^10^ The mechanism of this condition is explained as the release of histamine from mast cells, triggering H1 and H2 receptors in heart chambers and coronary vessels, causing vasospasm, a lack of sufficient blood supply to the heart, and consequently, the emergence of the IHD symptoms.^11^ Several triggers have been discussed as causes of Kounis syndrome (such as foods and bee stings), but the most prominent ones are antibiotics and nonsteroidal anti‐inflammatory drugs (NSAIDs), antineoplastic drugs, contrast media, corticosteroids, and proton pump inhibitors.^11^, ^12^, ^13^

In this case, we present a 51‐year‐old man without any past medical history (PMH) who presented to the emergency department (ED) with a complaint of chest pain and palpitations an hour after receiving an intramuscular (IM) injection of diclofenac.

## 2 CASE PRESENTATION

Case history/examination: A 51‐year‐old male with no previous medical history was presented to the emergency department complaining of a recent onset of retrosternal chest pain, breathlessness, and rapid breathing. The patient's medical history included an allergy to penicillin and a positive family history of coronary artery disease. The patient reported having a 5‐day pain located at the lumbar area, and he had received an intramuscular injection of diclofenac 30 min before the initiation of the chest pain. Thirty minutes after the injection, his symptoms started with generalized purities, a rash, and then a feeling of difficulty breathing along with central heaviness in his chest. He had never had an allergic reaction to medication or food before, except for the mentioned allergic reaction to an IM injection of penicillin. His wife was a general physician, and when she saw the symptoms, she injected IM chlorpheniramine, which led to the subsidence of his pruritis and rash. However, the chest pain and difficulty breathing were not responsive to the treatment. In his general appearance, anxiousness and diaphoresis were obvious. However, he was conscious and responsive to questions. His vital signs assessment showed tachycardia (HR = 130/min) and tachypnea (RR = 25/min), oxygen blood saturation of 94% when breathing ambient air, and high blood pressure of 160/95 mmHg and normal temperature (T: 36.8°C). In his chest examination, normal sinus tachycardia and rapid breathing were evident. No other remarkable sign was detected, and breathing sounds were normal.

**FIGURE 1:**
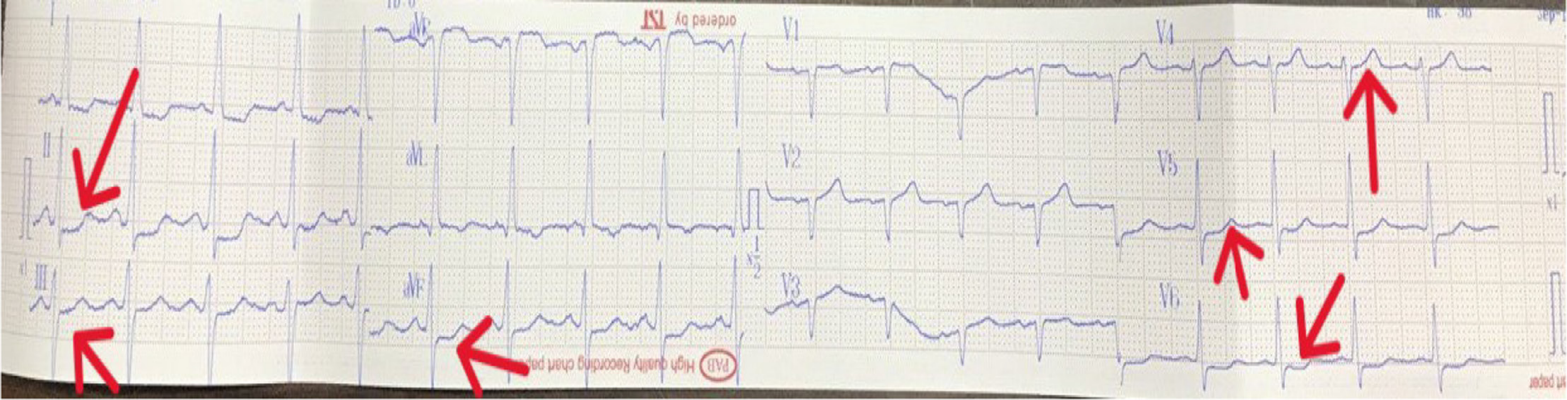
ST‐segment depression in leads I, II, III, AVL, AVF, and V5‐V6 precordial leads was detected in his initial ECG in the emergency department. Moreover, the ST‐elevation can be seen in lead AVR.

**FIGURE 2:**
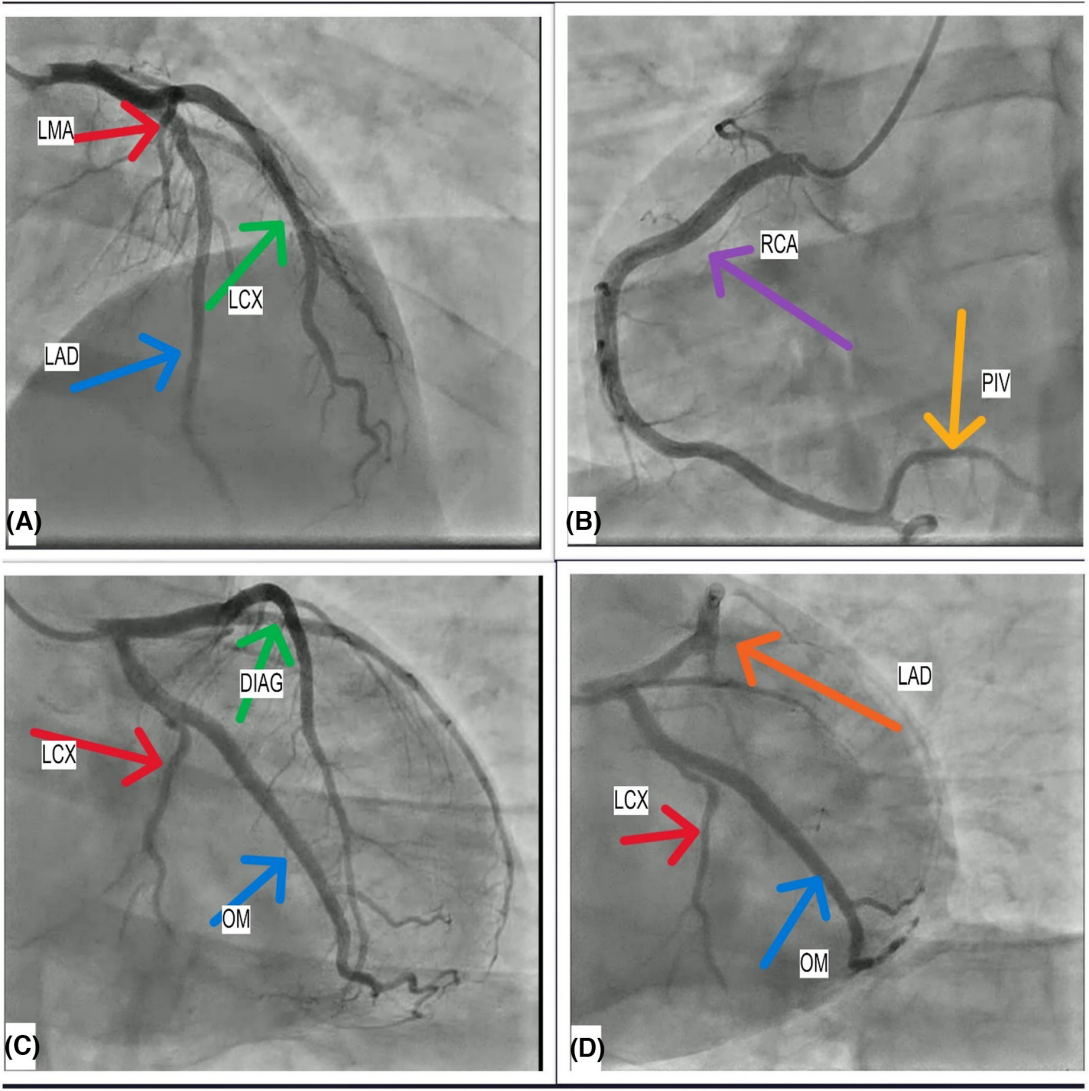
Coronary angiography (CA) of the patient shows no significant lesion on (A) left main (LMA), left anterior descending (LAD), and left circumflex (LCX) arteries. (B) No significant lesion at the proximal portion, right coronary artery (RCA), and posterior interventricular artery (PIV). (C) No significant stenosis in diagonal (DIAG); left circumflex and obtus marginal (OM) arteries. (D) No significant lesion on, lateral anterior descending; left circumflex, and obstus marginatum arteries.

Methods: Immediately after a physical examination, an electrocardiogram (ECG) was performed, which showed ST depression in leads I, II, III, AVL, AVF, and V5‐V6. In addition, ST‐segment elevation was detected in the AVR lead (Figure 1). The echocardiogram revealed no significant abnormality. The LVEF was 65%, and no abnormality was seen in his heart's motion, and no abnormality was seen in his heart wall motion. High‐sensitivity cardiac troponin and Creatine phosphokinase‐MB were checked (Table 1). His heart rhythm and rate, along with oxygen saturation and respiratory rate, started to be monitored immediately. Suspecting acute coronary syndrome (ACS), serial ECGs were started, and administering aspirin (325 mg), clopidogrel (300 mg), atorvastatin (80 mg), nitroglycerin (10mcg per minute intravenous), and an unfractionated heparin (UFH) infusion (60 U/kg/h) and metoprolol 50 mg orally was given to the patient. Despite receiving medication, the chest pain persisted for 12 h and did not decrease. His ECG changes remained persistent, and no arrhythmias were observed. The initial and the second troponin level tests (after 6 h) were within the normal range. A transthoracic echocardiogram revealed a normal ejection fraction with no regional wall motion abnormalities. Due to the ST‐segment changes on ECG, a coronary angiogram (CA) was performed, revealing normal coronary arteries (Figure 2).

**TABLE 1:** Laboratory findings of the patient.

Test	Result	Reference range
RBC (10^6^/μL)	5.06	4.2–5.5
Hemoglobin (g/dL)	14.3	12–16
WBC (per μL)	5920	4.000–11.000
MCV (fL)	85.8	80–99
Hematocrit (%)	43.4	37–47
Platelet (per μL)	206.000	150.000–400.000
Neutrophils (%)	81%	40–75
Lymphocytes (%)	14.6%	20–45
Eosinophils (%)	4.4%	0–6
Lipid profile, coagulation factors, and troponin		
Cholesterol	151	Up to 200
TG (mg/dL)	44 mg/dL	Up to 150
LDL (mg/dL)	74 mg/dL	Up to 130
HDL (mg/dL)	53 mg/dL	>45 mg/dL
Blood Sugar (mg/dL)	96 mg/dL	74–106 mg/dL
LDH (mg/dL)	325 Iu/L	235–470 Iu/L
K^+^ (meq/L)	4	3.5–5.3 meq/L
Creatinine (mg/dL)	0.95	0.5–1.00
Urea (mg/dL)	28	13–43
Hb A1C %Hb	5.8	4.8–5.9
PT	15.3 s	11–13 s
PTT	31 s	25–38 s
INR	1.31	1–1.5
CK‐MB	15 μ/L	<25 μ/L
Troponin I	Negative	Negative

*Note*: *Urine analysis*: No remarkable pathologic finding was detected.

Outcomes and follow‐up: He was followed up in the cardiology ward for 24 h. After repeating the ECG several times, the initially observed ST‐segment depressions were gradually reduced and completely disappeared after 12 h. The chest pain and difficulty in breathing resolved as well. Over 12 h, the patient's ECG gradually returned to normal, and the changes were completely resolved. The serial echocardiogram showed a normal left ventricular ejection fraction and no significant valvular abnormality. Serial troponin levels were normal. Considering the mentioned dynamic changes in the patient's ECG, normal troponin level, and lack of any pathologic finding in his CA, the diagnosis of unstable angina was made for the patient.

**FIGURE 3:**
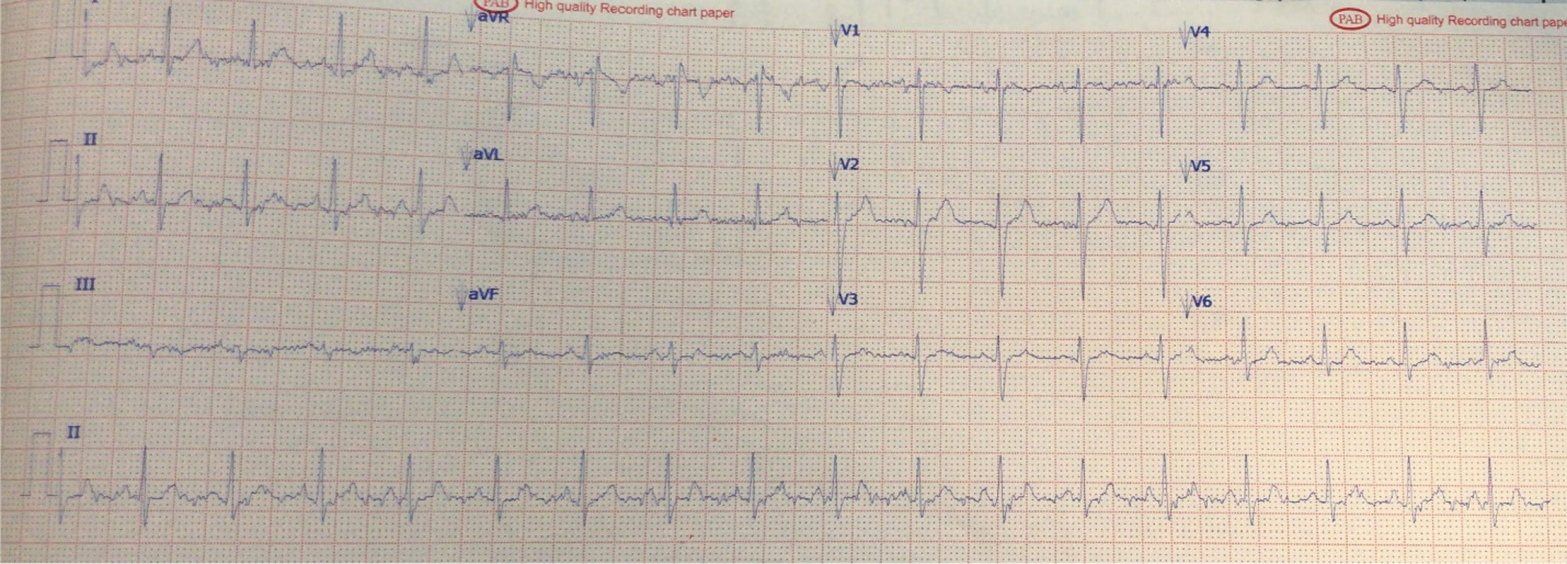
One day after the coronary angiography, the abnormalities on the patient's ECG were resolved.

The day after the (CA), the symptoms were completely resolved, and the physical examination revealed normal heart rate and rhythm, respiratory system function, and neurological function. The pathologic changes in the patient's ECG were resolved (Figure 3). A good clinical outcome was confirmed at a follow‐up visit 6 months later. He was advised not to take diclofenac again and was referred for a drug allergy evaluation.

## 3 DISCUSSION

In this case report, we presented a 51‐year‐old man without significant PMH (except being allergic to penicillin) or FH who presented with typical retrosternal chest pain after an intramuscular injection of diclofenac. It has been shown that Kounis syndrome is mainly caused by inflammatory mediators, which are released during allergic insults, post‐inflammatory cell activation, or when multidirectional stimuli cause interactions. A platelet subset of 20% with high‐ and low‐affinity IgE surface receptors is also involved in this process.^14^ Clinically, it is defined as the concurrent occurrence of allergic reactions, anaphylactic shock, hypersensitivity shock, and acute coronary syndrome (ACS). This syndrome is categorized into three subgroups: (1) Type I is seen in patients with normal or nearly normal coronary arteries without any predisposing factors for coronary artery disease (CAD). Although the release of mediators mostly causes vasospasm in coronary arteries without any rise in cardiac enzymes, some of them have more progression and might cause acute myocardial MI (AMI). (2) Type II: the histamine release in patients with preexisting plaque atheroma in coronary vessels. It can cause spasms without cardiac enzyme rise or, in more severe cases, MI with erosion or rupture of the plaque. (3) Type III: patients with thrombosis of the drug‐eluting stent, accompanied by mast cells and eosinophils.^7^, ^15^ Type III Kounis was classified at IIIa, known as stent thrombosis, and IIIb, which is named restenosis.^8^, ^16^

Kounis syndrome can occur at all ages; however, men between the ages of 40 and 70 are more vulnerable to this complication. Moreover, some geographical distributions are more widely seen in reported cases of this syndrome. One of the difficulties in the diagnosis of this syndrome is the unawareness of the physicians of this syndrome and their failure to associate drug use with cardiovascular symptoms. This relatively rare syndrome and its mechanism are not well understood. However, it is hypothesized that the release of histamine from mast cells and platelet reactivation, which occur due to exposure to triggers, occur either as a result of antigen binding to IgE antibodies on the mast cell surface or as a consequence of complement system activation.^7^, ^11^ These histamines and inflammatory mediators activate H1 and H2 receptors in the heart muscle and coronary vessels and cause the narrowing.^15^, ^17^ Some studies concluded that mediators with the potential to cause vasoconstriction, such as leukotrienes, chymase, and cathepsin D, have an active role in this process.^18^

NSAIDs, including diclofenac, are one of the most recognized allergen medications. They induce this reaction through two main mechanisms. (1) Drug‐specific IgE antibodies in hypersensitive patients to that antibody. (2) Pharmacologic action of this group of drugs because they inhibit cyclooxygenase and stimulate the lipoxygenase pathway of arachidonic acid metabolism, increasing leukotriene production.^19^, ^20^, ^21^ One of the most important points is the high prevalence of previous drug allergies among patients who experience this condition (25%). However, these reactions might be seen in patients who have previously received these medications and have shown no specific response, specifically in those who have received the medication IM or IV.^11^ Kounis patients are presented mainly with two types of symptoms. First, mild, moderate, or severe allergic reactions; and second, cardiac symptoms. The symptoms are mostly seen within an hour after the administration, and the mean time of onset of symptoms is shorter in beta‐lactamases compared to NSAIDs. Hypotension and chest pain are the most common cardiovascular symptoms, and GI, respiratory, hypotension, and dermatologic are seen less commonly compared to the mentioned two cardiovascular symptoms.^22^, ^23^

Diagnosis is based on an accurate history, physical examination, laboratory data, electrocardiographic data, echocardiographic data, and angiographic evaluation.^24^ First, asking about the history of any drug allergies is crucial. In laboratory data assessment, serum tryptase, histamine,^25^ cardiac enzyme, and cardiac troponin levels may be helpful as indicators of hypersensitivity, as well as cardiac enzymes such as troponin, creatine kinase, and creatine kinase myocardial band, as they show myocardial injury.^26^ ECG changes such as ST‐segment elevation or depression are indicators of myocardial ischemia. ST‐elevation MI is more commonly seen in Kounis syndromes due to NSAIDs and beta‐lactam antibiotics.^22^ Echocardiography (transthoracic) is mostly utilized as a diagnostic procedure to rule out differential diagnoses such as dissection of the aorta.^27^ The catheterization is useful for the differentiation of stenosis from spasm.^25^

Treatment of Kounis syndrome is based on the type of syndrome. In Kounis syndrome 1, treatment would result in the disappearance of all symptoms. However, in Type II, both allergic and cardiac symptoms should be approached simultaneously. For an allergic person, hydrocortisone 1–2 mg/kg, ranitidine, or diphenhydramine would be mostly enough. Repletion of the fluid due to increased permeability is crucial, and treatments, including vasospasm, such as calcium channel blockers, should also be considered. However, the fluid infusion should be done cautiously as the left ventricle function might deteriorate due to diminished supply. Oxygen supplementation is crucial in the treatment process for all patients.^15^, ^26^, ^27^ Notably, adrenalin use should be considered more thoughtfully as it can cause worsening of the vasospasm. Its additives can also cause allergic reactions. Morphine, codeine, and meperidine have the potential to worsen the allergic reaction and should be avoided. Acetaminophen can also lead to hypotension and is not recommended for pain control. On the other hand, fentanyl is a safe choice to alleviate the pain caused by vasospasm.^15^, ^25^, ^28^ Kounis syndrome type III should be treated, as should ACS patients. Overall, the prognosis of chest pain due to Kounis syndrome is much better than that of coronary vessel issues. It is estimated that Kounis syndrome has a mortality risk of about 2.9% of cases.^25^

Many medications, especially NSAIDs and antibiotics, have the potential to cause allergic reactions. This reaction might be seen accompanied by or only with cardiac symptoms (like ACS). Since this incidence is more prevalent in patients with drug allergies, the history should be taken carefully before prescribing medication. Any cardiac complaint, especially in a short time after drug administration and with allergic symptoms, might be due to Kounis syndrome. These patients' heart conditions should be evaluated as if they have cardiac disease because the vasospasm caused by these reactions can lead to myocardial infarction and even death. Fluid repletion, analgesic administration, and nitrate use must be done considering several factors different from normal ACS patients.

## AUTHOR CONTRIBUTIONS

**Pouya Ebrahimi:** Conceptualization; data curation; formal analysis; project administration; resources; writing – original draft; writing – review and editing. **Roozbeh Nazari:** Conceptualization; data curation; formal analysis; methodology; project administration; resources; writing – original draft; writing – review and editing. **Nahid Senobari:** Conceptualization; data curation; formal analysis; methodology; project administration; resources; writing – original draft; writing – review and editing. **Seyedeh Maryam Mousavinezhad:** Funding acquisition; investigation; software; supervision; validation; visualization; writing – review and editing. **Delaram J. Ghadimi:** Investigation; methodology; software; supervision; validation; writing – review and editing. **Hamidreza Soleimani:** Investigation; methodology; software; supervision; validation; writing – review and editing.

## FUNDING INFORMATION

No funds were received for this study.

## CONFLICT OF INTEREST STATEMENT

The authors declare no conflicts of interest.

## CONSENT

Written informed consent was obtained from the patient to publish this report in accordance with the journal's patient consent policy.

## Data Availability

Data are available on request due to privacy/ethical restrictions.
